# Navigating NTRK in Non-small Cell Lung Cancer (NSCLC): A Patient Perspective

**DOI:** 10.7759/cureus.111342

**Published:** 2026-06-23

**Authors:** Justin Gainor, Leah Bundy, Stevan Bradford

**Affiliations:** 1 Center for Thoracic Cancers, Massachusetts General Hospital, Boston, USA; 2 Medicine, Springer Health+ IME, London, GBR; 3 Patient author, N/A, Calvert, USA

**Keywords:** biomarker testing, non-small cell lung cancer (nsclc), ntrk fusion, patient perspective, podcast, targeted therapies

## Abstract

*NTRK *fusion-positive non-small cell lung cancer (NSCLC) represents a rare molecular subtype in which effective clinician-patient communication and an understanding of the lived experience are essential to supporting personalized care. This podcast presents a video interview exploring experiences associated with *NTRK* fusion-positive NSCLC, examining key stages of the diagnostic and treatment pathway, including biomarker testing, the emotional impact of diagnosis, initiation of targeted therapy, and managing treatment-related adverse events. It also considers the psychological impact of *NTRK* fusion-positive NSCLC, including the emotional burden of diagnosis, challenges of independently seeking information about rare alterations, treatment delays, and the transition from chemotherapy to a targeted therapeutic approach. Emphasis is placed on the importance of timely biomarker testing and transparent communication around biomarker testing and treatment timelines, as well as greater clinical recognition of the uncertainty often experienced by individuals with rare genomic alterations. By amplifying the patient voice, this piece seeks to foster greater awareness and empathy among healthcare professionals and contribute to improved outcomes in precision oncology.

## Editorial

This submission is a transcript of an interview between an expert oncologist and a patient with *NTRK* fusion-positive non-small cell lung cancer (NSCLC) (Video 1). 

**Video 1:** Navigating NTRK in NSCLC: a patient perspective. NSCLC, non-small cell lung cancer

Chapter 1: Introduction and NSCLC diagnosis from the patient perspective

[Justin Gainor: 00:04.5] Hello. My name is Dr. Justin Gainor. I'm a Thoracic Medical Oncologist and Director of the Center for Thoracic Cancers at the Massachusetts General Hospital and an Associate Professor of Medicine at Harvard Medical School. Today I have the pleasure of being joined by Mr. Stevan Bradford. Stevan, I'll ask you to introduce yourself.

[Stevan Bradford: 00:26.0] Hello, I'm Stevan Bradford. I'm living in Alabama in the Mobile area. I work in a steel mill. I learned in January that I was diagnosed with stage four non-small cell lung cancer.

[Justin Gainor: 00:42.7] Well, we're really grateful that you're taking the time today to share your experience with us. And maybe you could tell us a little bit more about how you were diagnosed back in January.

[Stevan Bradford: 00:58.1] Yes, in January I was just visiting my primary care doctor for just a checkup and to get my prescriptions recertified and reauthorized. During that checkup, the doctor noticed a lump on my left side clavicle area. And that led to a chest X-ray and then to a CT scan.

[Justin Gainor: 01:27.9] Oh, I'm sure that was a surprising and overwhelming experience. Kind of just going based on a primary care visit.

[Stevan Bradford: 01:38.8] Yes. I wasn't feeling any type of pain, wasn't having any coughs, and it was just kind of a shock. Of course, here I am going to start researching what was being said, which led to some information that I was still waiting for a diagnosis, but now I've already had some information that I've gathered which was kind of interesting but depressing.

Chapter 2: Biomarker testing and emotional impact of finding a targetable *NTRK* fusion

[Justin Gainor: 02:08.7] Maybe you could share a little bit more with us. When you made it to the oncologist after that testing, what did they relay to you? You know, when they gave you this diagnosis?

[Stevan Bradford: 02:24.2] Yes, on my first meeting with my oncologist, he said that they had done a tissue sample. We have learned that you do have stage four adenocarcinoma, and he was discussing with me that it's not curable, but it's treatable, and that we would be trying to send off some tissue and blood for some biomarker testing.

[Justin Gainor: 02:53.4] Okay, so it sounds like even from that first visit they were talking to you about biomarker testing. What were your expectations? Did they give you a sense of how long it would take and why it was important?

[Stevan Bradford: 03:11.5] They said it was important to try to find a targeted therapy for me. Otherwise, I probably was looking at two to three years on the scheme of things. So we were pretty interested and of course, I left there researching biomarkers and what they may lead to.

[Justin Gainor: 03:32.9] And you mentioned tissue testing, so that would be testing the tumor. I'm curious at the time, did they introduce or mention liquid biopsies as well?

[Stevan Bradford: 03:48.4] Yes, the liquid biopsy had come back within a short time, and it didn't show any biomarkers were present in the blood biopsy. But he was encouraging me that it’s not 100% accurate, that the tissue sample would be the real defining test, and it would take another three or four weeks to get that back.

[Justin Gainor: 04:16.0] And you're here today and you had shared with me before, but maybe for our audience, what did those biomarker tests come back showing?

[Stevan Bradford: 04:27.9] I had read the report, but I was having a hard time understanding a lot of it. But I did look at some of the present characteristics of my tissue sample. It did mention that I had a *NTRK1* fusion, and I didn't know what that was. So we started looking into that and we weren’t finding very much information about it. I sought a second opinion before my biopsy for [biomarkers] and both doctors did say I was looking at starting chemotherapy, and I did have one chemotherapy treatment. Not the full treatment, just one session.

[Stevan Bradford: 05:23.6] And then, before my next chemotherapy treatment - which were every three weeks - at the end of those three weeks before my next treatment, we found out that I did have *NTRK* fusion‑positive NSCLC. So we stopped the chemotherapy and started looking forward to starting a targeted therapy.

[Justin Gainor: 05:47.8] And I'm sure that your doctors relayed that this is good news, right? To find a targetable alteration. As an oncologist, we're always excited when we find things like this.

[Justin Gainor: 06:06.3] But you've already touched on this. This can also be an emotional rollercoaster, right? While you're waiting for results and you start down one treatment pathway and then pivot towards something else. I'm curious, what support did you find most helpful, particularly early on in your treatment course?

[Stevan Bradford: 06:31.3] Early on I found a few groups on social media that were kind of helping me get through because it was a fast-paced rollercoaster that I seemed to be on. Also, it seemed like it was taking quite a bit of time to actually get started on a treatment.

[Stevan Bradford: 06:50.2] So it kind of put me in a not a very good place, knowing what was coming and what may happen, and that kind of thing. But I was happy to learn that I did have a targetable biomarker.

Chapter 3: Understanding rare biomarkers and targeted therapies as a patient

[Justin Gainor: 07:08.8] When you went into that first visit where they're relaying the genotyping results, what do they tell you about NTRK and targeted therapies? And what you might experience when you start on these drugs?

[Stevan Bradford: 07:30.8] It was almost like me and my oncologist were learning this for the first time. He wasn't really up to date on my specific biomarker and what my therapy would be. And it was kind of like, we were excited to start it, but we were also both looking for information. I left the office wondering how this might go. I started of course trying to research it for myself, how the different therapies would work for me, and was finding out that in some cases there were really good results and then most of them were showing about at least a 27 month [response], a good positive result and then some even further [1,2]. But it was also a very new therapy and there wasn't a whole lot of information out there.

[Justin Gainor: 08:36.6] Yeah, I think you raise really good points about the rarity of *NTRK* fusion-positive NSCLC [3], and as a result, many practitioners haven't seen patients with this alteration or haven't used some of these agents before. I can imagine this creates a lot of trepidation of that uncertainty. What resources did you find most helpful when you said that you were researching things? Were there particular resources that you found really helpful?

[Stevan Bradford: 09:15.4] I found a few pretty good videos on the subject and besides mentioning that it was rare, there were some good results coming out of this area. So I was positive in starting this therapy.

Chapter 4: Initiation of a targeted therapy and early treatment experience

[Justin Gainor: 09:33.6] Great. It sounds like you then started on a targeted therapy. Maybe you can tell us a little bit about what your experience has been and whether there have been any side effects since starting it?

[Stevan Bradford: 09:48.4] Yeah, when I first started the targeted therapy, I was having some side effects in the beginning with muscle pains. It seemed like each day it might be in a different area. I was actually using compression socks and some massage machines too.

[Stevan Bradford: 10:11.0] It mainly was affecting me in the evening time. That seemed to last about two and a half weeks and they kind of just went away. After that, I did start having lightheadedness, dizzy spells, and had a few occurrences of passing out, which led to me to stopping my blood pressure medicines. After that I haven't passed out again. I still get a little lightheaded, but it's manageable.

[Justin Gainor: 10:47.4] And maybe you could tell us, the dizziness, was that early on in your course or was it later?

[Stevan Bradford: 10:58.4] It seemed to come a month after starting the targeted therapy.

[Justin Gainor: 11:04.0] Okay.

[Stevan Bradford: 11:04.7] Just gradually getting worse. As soon as I stopped my blood pressure medicine, it got to where it was only upon standing, to where I just take a minute before I continue because I get a little lightheaded. It's still ongoing.

[Justin Gainor: 11:28.2] Well, I'm glad. It sounds like it's gotten better since the discontinuation of the blood pressure medicine. So that's great. How often were you seeing your oncologist during this time?

[Stevan Bradford: 11:44.6] I believe it was every three to four weeks. And this all started in January. By February, I was well on my way. Well, February was a time when everything did just seem to take too long to get started. We were feeling like I need to start treating this, because I didn't understand at the time that it just takes a little while to get a patient lined out on the treatment. But it kind of had me feeling like I was in a rush to start something, you know, to fight what I was experiencing.

Chapter 5: Treatment response and impact

[Justin Gainor: 12:37.3] And, which agent are you on now?

[Stevan Bradford: 12:44.3] I had my third PET scan here just a few weeks ago. So, I started taking my TRK inhibitor on March 5th. On April 6th, I had my second PET scan, and it showed a great deal of improvement in my condition to where, most of my lymph nodes were getting back to normal and not really showing a lot of the metastasis that had shown up before.

[Justin Gainor: 13:23.5] That's fantastic. That's great news. And it sounds like now, from a side effect profile, you're also in a better place. It sounds like the muscle aches have resolved and the dizziness also seems like it's in a better place.

[Stevan Bradford: 13:44.9] Yeah, I went from thinking about retirement when I first got diagnosed, and after taking the targeted therapy and feeling better, I actually went back to work and have been back at work for about two months.

[Justin Gainor: 13:59.9] That's terrific. I imagine that's also very different to what you probably envisioned when you were first given the diagnosis, seeing that you can treat lung cancer like this, with a pill.

[Stevan Bradford: 14:16.3] Yeah, that's right. It was all new to me. My dad had cancer, esophageal. And he passed away in about a year and a half. So my mind was all on let's take care of what I need to take care of, before I get too bad off to where I'm not helpful.

Chapter 6: Conclusions, patient reflections, and advice for clinicians practicing precision medicine

[Justin Gainor: 14:40.8] Yeah. Well, I'm glad that things are going so well. Maybe to wrap things up, there will be a lot of physicians and medical professionals listening to this. I'm curious whether you could share with us what are some of the things that you wish more doctors understood about your personal experience with this diagnosis and the journey to this point?

[Stevan Bradford: 15:09.8] Well, I knew from being a non-smoker that I seemed to have a good chance of finding some type of biomarker to treat. And hearing that I was stage four and it was not curable but treatable, and I had a prognosis of two to three years, it was kind of a shot that I didn't really feel too good about. And there was just a little mention of the biomarker testing to find something we could treat. It seemed like it was just a footnote compared to the initial beginning of my treatment and diagnosis.

[Justin Gainor: 15:59.0] I see. It sounds like you'd have preferred some more emphasis on the biomarker piece up front in terms of likelihood and what specifically they'd be looking for.

[Stevan Bradford: 16:11.4] That's right. And I do realize that this is a fairly new area that is advancing lately and it's even getting more attention than it had in the past. It seems like there's lots of gains being made every day in this area.

[Justin Gainor: 16:34.2] You're exactly right. I think tremendous progress and I think you're a testament to that. Any closing pieces of advice for clinicians or patients like yourself, as they're being faced with a diagnosis like this?

[Stevan Bradford: 16:57.1] I just know it seemed amazing to me to be at stage four and only be on a targeted therapy for maybe four weeks and to show the improvement that it did. I can’t get my head around how fast that worked, and what's going on inside the cells, and what the difference between a fusion and mutation is. I'm still learning every day the differences of what I have. And the future also. I think I'm going to be taking the targeted therapy until it shows that it's not really working, and then we'll adjust to maybe a combination of a couple other ones. I'm just learning every day and realizing that it's all fairly new.

[Justin Gainor: 17:53.0] Well, I think you're a great example of a story of hope and as you mentioned it's a journey and doing one's own research, learning along the way, talking to your clinical team, I think these are all great points. And it really underscores the importance of molecular testing because finding an alteration like an *NTRK* fusion can be a game changer. I really want to thank you for taking the time and sharing your story with us today. Thank you.

[Stevan Bradford: 18:30.0] Thank you.
